# Grouped data with survey revision

**DOI:** 10.1186/s12874-023-01834-7

**Published:** 2023-01-16

**Authors:** Chung-Han Liang, Da-Wei Wang, Mei-Lien Pan

**Affiliations:** 1 Department of Computer Science and Information Engineering, National Taiwan University, Taipei, Taiwan; 2 Institute of Information Science, Academia Sinica, Taipei, Taiwan; 3 Information Technology Service Center, National Yang Ming Chiao Tung University, Taipei, Taiwan

**Keywords:** Survey revision, Data preprocessing, Data cleaning, Grouped data, Stochastic process, Matrix decomposition

## Abstract

**Introduction:**

Surveys are common research tools, and questionnaires revisions are a common occurrence in longitudinal studies. Revisions can, at times, introduce systematic shifts in measures of interest. We formulate that questionnaire revision are a stochastic process with transition matrices. Thus, revision shifts can be reduced by first estimating these transition matrices, which can be utilized in estimation of interested measures.

**Materials and method:**

An ideal survey response model is defined by mapping between the true value of a participant’s response to an interval in the grouped data type scale. A population completed surveys multiple times, as modeled with multiple stochastic process. This included stochastic processes related to true values and intervals. While multiple factors contribute to changes in survey responses, here, we explored the method that can mitigate the effects of questionnaire revision. We proposed the Version Alignment Method (VAM), a data preprocessing tool, which can separate the transitions according to revisions from all transitions via solving an optimization problem and using the revision-related transitions to remove the revision effect. To verify VAM, we used simulation data to study the estimation error and a real life MJ dataset containing large amounts of long-term questionnaire responses with several questionnaire revisions to study its feasibility.

**Result:**

We compared the difference of the annual average between consecutive years. Without adjustment, the difference is 0.593 when the revision occurred, while VAM brought it down to 0.115, where difference between years without revision was in the 0.005, 0.125 range. Furthermore, our method rendered the responses to the same set of intervals, thus comparing the relative frequency of items before and after revisions became possible. The average estimation error in L infinity was 0.0044 which occupied the 95% CI which was constructed by bootstrap analysis.

**Conclusion:**

Questionnaire revisions can induce different response bias and information loss, thus causing inconsistencies in the estimated measures. Conventional methods can only partly remedy this issue. Our proposal, VAM, can estimate the aggregate difference of all revision-related systematic errors and can reduce the differences, thus reducing inconsistencies in the final estimations of longitudinal studies.

**Supplementary Information:**

The online version contains supplementary material available at 10.1186/s12874-023-01834-7.

## Introduction

### The issue of survey revision

Researchers often use questionnaires as a tool to measure values that are difficult to obtain simply through observations. These values are mostly related to people’s habits or thoughts. Psychologists usually use various psychometric properties to describe the quality of the questionnaire, which can be roughly divided into two types, reliability (having consistent measurements) and validity (it measures what is supposed to be measured) [1]. Survey methodologists usually care more about measurement error, which can be divided into two categories, random error and systematic error (or bias). The difference between the two is that random error can decrease when the amount of data is large enough, while a systematic error can continue to exist.

In conducting long length survey research, two issues may arise, response burden and out-of-date statements and response options. It is reasonable to use closed-ended questions to conduct survey research when there is a large number of items to be answered [2], but closed-ended questions may introduce information loss [3]. Most interpretation methods have not considered information loss appropriately. This can lead to overestimations or underestimations of the underlying numerical answers. This would lead to being categorized as a systematic error. The two examples below show how using naive methods may lead to results with systematic errors. The first example is top-coded data, which commonly appears in closed-ended questionnaire responses, with an unknown underlying distribution that is greater than a specified bound. Suppose that there is a random variable *X* that denotes the numerical answer in the respondent’s mind and a top-coded response option with lower bound *a*. As the upper bound of the top-coded response option is infinity, it is quite naive to choose the lower bound as the expected value of the response option. This method underestimates the expected value. The second example is grouped data, which is commonly obtained by closed-ended questionnaires. This example can easily lead to overestimations and underestimations because the distribution in each group is unknown. Given a random variable *X* denotes the answer in the respondent’s mind and follows a truncated normal distribution with $$\mu$$=0 and the distribution lies within $$[10^{-6}, 10^{6}]$$. A closed-ended questionnaire separates the truncated normal distribution by a bound $$b(b\ge 10^{-6}, b\le 10^{6})$$. This means that there are only two response options, one corresponding to the interval $$[10^{-6}, b)$$ and the other corresponding to the interval $$[b, 10^6]$$. It is intuitive to assume the answer in mind in each option is uniformly distributed and the midpoint is used as the mean of that option. In this example, the only chance that the naive midpoint method would not lead to over or underestimations of the expected value of *X* is setting $$b=0$$.

During long-term survey response collections, a questionnaire revision may occur to keep the survey statement or response options up-to-date. Different versions of closed-ended questions introduce different kinds of information loss. The estimation by a naive method which contains different systematic errors implies an inconsistency between the estimated result. However, when the adjustment in revisions is small, we assume that the inconsistencies can be recovered by other information in the data. In practice, it is common to screen responses from a single version of a questionnaire during data preprocessing, to avoid interference or inconsistencies induced by multiple versions of the questionnaire. Discarding a large amount of data may cause biased analysis results, thus, analyzing data from different versions of a questionnaire jointly, is reasonable. To our best knowledge, there is no research aimed at solving inconsistencies between responses from different versions of a questionnaire. Hence, we propose a method that may reduce such inconsistencies.

Two types of closed-ended questionnaires are commonly used. One is the Likert-type scale and the other is the grouped data type scale. A Likert-type scale is commonly used in collecting data with regards to sensation, for example, whether a software program is helpful [1]. Another common type of closed-ended scale is grouped data type scale, and this is often used to collect a continuous numerical answer from respondents. In this study, we focus on the revisions that occur on the grouped data type scale. A grouped data type scale asks for a specific objective number and provides some interval as options. Moreover, revisions on grouped data type scales often preserve the statements of the questions, and only adjust the upper and lower bound of intervals in options. This can infer that the participant’s perception of the question does not change much after revision. Due to this property of grouped data type scales, we propose that it is possible to describe the changes of the responses due to questionnaire revision through a probability model.

### Problems with grouped data type scale revisions

Data collected by grouped data type scales is grouped data and grouped data are commonly used in various fields. For example, research on income inequality usually relies on grouped data [4]. One reason for using grouped data is to protect personal privacy, such as those with the highest income, or whose identity can be deduced easily. Individual income observations are classified into one of several income brackets, and so researchers can only observe the number of individuals belonging to each bracket. Without information on the distribution within each bracket, specific identities within the group are kept hidden. Another example of observing grouped data is sieve analysis in civil engineering [5]. A sieve analysis separates particles by different sieves with different diameters. Through multiple sieves, the proportion of particles in different size ranges can be obtained, which reveals the particle size distribution of the whole mass.

It can be found that one reason behind collecting grouped data is that some restrictions are placed on data collection, since the individuals are divided into multiple categories and each category denotes a numerical interval. Grouped data can be treated as ordinal data when considered as a rating task, however, information is lost when it is provided by the numerical intervals. On the other hand, even considering the numerical intervals, analyzing grouped data can pose a big challenge, such as the open-ended top bracket or top coded data problem. The top coded data belongs to the top interval, which has an unlimited upper bound. It is difficult to estimate its statistics and can cause systematic errors when inappropriate analysis methods are used.

When using grouped data type scales to conduct a self-report study, every participant may have a different perception of the numerical interval belonging to each option, thus, the variance of bounds belonging to each interval is another problem while analyzing grouped data. Liddell, T. M. et al. [6] proposed that even Likert-type scales do not provide the numerical intervals, participants will still give each option an interval they believe that is appropriate, thus, taking account of the unknown bounds during analysis is necessary. Similarly, grouped data collected by a self-report scale shares this same property, which is that the responses are categorical. Additionally, the extra information of the intervals that grouped data provides can be considered as an unreliable reference. Thus, it can be analyzed by the method that is proposed by Liddell, T. M. et al. [6].

The difference between each participants’ perception of the interval bounds and the bounds that the questionnaire provides can be considered a random error. As mentioned before, random error converges to 0 when the sample size is large. Then, it’s reasonable to use the Maximum Likelihood Estimator (MLE) proposed by Xiao, X. et al. [5] with the bounds that the questionnaire provides to analyze grouped data. Nonetheless, when it comes to questionnaire revisions, inconsistencies within the information loss are still not considered and the estimated underlying distribution statistic still contains unexpected errors. In this study, participant’s perceptions of the bounds were considered as random errors and grouped data was analyzed via MLE, since the information of bounds was assumed to be reliable.

In this study, the changes in grouped data type survey responses were modeled via a discrete state stochastic process, since the responses were categorical which can be represented by states, and the shift between response options can be represented by transitions between states. A previous study by Chiba, T. et al. [7] modeled the changes in consumer preference as a Markov chain and represented them by a transition matrix. Additionally, they proposed that consecutive transition matrices are similar and they can use l1-norm to measure the distance between them. These intuitions helped model the changes in survey responses. However, the assumptions made about the Markov chain which included the transitions that were sufficiently high, implied the stationary observation and the states in each time were fixed, did not fit the survey revision scenario. Thus, we propose our own method to estimate the transitions in the stochastic processes.

### Specific aims

To our best knowledge, there is a lack of research on how to analyze the data collected by different versions of a questionnaire. At the same time, grouped data type scales are often used in a longitudinal study on self-reported health questionnaires [8], but it contains various information loss that can cause data inconsistencies when questionnaire revision occurs. Thus, our aim is to reduce the revision-related inconsistencies of systematic errors by aligning the implicit difference in responses induced by revisions. The implicit difference includes information loss and response bias.

We proposed the Version Alignment Method (VAM) to align grouped data collected by different versions of a questionnaire. In VAM, we considered the changes in grouped data over time as transitions of states, since each group can be regarded as a state. The change in response options by different source factors were modeled as different transitions. The transitions can be represented by transition matrices, and VAM estimates the transition matrices composed of transitions that represent a single factor via matrix decomposition, for example, regarding questionnaire revisions or the changes of underlying distribution. Following the matrix decomposition, the matrix which consists of revision transitions can be used to align the version of the questionnaire in grouped data. Matrix factorization is a similar topic, and it has been extensively used in recommendation systems to find relationships between variables, which can be solved by formulating it as an optimization problem. Unlike the recommendation system, the result of our matrix decomposition must satisfy the definition of the transition matrix and some properties related to the data being analyzed, hence, some constraints according to that needed to be added to the objective function.

A simple example below demonstrates the novelty of the VAM, which is that the algined versions producting estimations with lower mean estimation error regardless the methods used. A question is used for accessing the usefulness of a technology tool in a questionnaire as an example. The first version of the question is: How many of the 10 functions provided by the tool do you use? The options are $[0-6]$ and $[6-10]$. Later, the options were changed to $[0-3]$, $[3-4]$, $[4-6]$, $[6-8]$, and $[8-10]$. Consider the underlying answer of the question follows truncated normal distribution $N(\mu =5, \sigma ^2=1)$ which lies in [0, 10]. The first version of the question $Q_1$ provide two response options and the corresponding interval set is $I_1=\{[0, 6), [6, 10)\}$. The second version of the question $Q_2$ provide five response options and the corresponding interval set is $I_2=\{[0, 3), [3, 4), [4, 6), [6, 8), [8, 10)\}$. Assume the sample size is 10000, and the frequency of responses to $Q_1$ is [8413, 1587] and responses to $Q_2$ is [227, 1359, 6828, 1573, 13]. Suppose that a correction by VAM is applied to the responses to $Q_1$ with some error, the frequency of adjusted responses is [277, 1359, 6728, 1573, 63]. The mean estimations by the midpoint method and MLE based on frequency of responses to $Q_1$ are 3.7935 and 3.3947. The mean estimations based on frequency of responses to $Q_2$ are 5.0365 by the midpoint method and 4.9999 by MLE. Additionally, the mean estimations based on frequency of responses to VAM adjusted responses to $Q_1$ are 5.039 by the midpoint method and 5.0092 by MLE.

VAM was considered as a data preprocessing method, that did not make too many assumptions about the dataset and did not use too much information, except the variable that needed to be adjusted or aligned, to let the effect on subsequent analysis remain small. Therefore, VAM only used cohort information for data alignment and an assumption on the underlying distribution of grouped data.

In this study we introduced The Survey Response Model and The VAM. The Survey Response Model defined an ideal process of an individual answering a grouped data type scale. The VAM leverages the Survey Response Model and models the effect of revision as transitions in stochastic processes. Then, it finally reduces the effect of revisions by estimating the transition matrix composed by the transitions related to revisions and applied it on the data.

The remainder of this paper is structured as follows. In the Materials and methods section, we first introduce how we model the ideal process of answering the grouped data type scale, then introduce the structure of VAM and the constraint of input/output data. At the end of this section, we introduce the two datasets we used for verification. The first one is the MJ dataset that has been used in many longitudinal studies. The next one is the simulation dataset, generated by the ideal process of answering a grouped data type scale, for observing the actual errors that can be induced by VAM. We list the results of using VAM on the MJ dataset and simulation dataset and verify that the results are reasonable in the Results section. We summarize the difference between using VAM and not, then list the conditions of a dataset to which VAM is applicable, and also propose some methods when VAM is not applicable to the dataset in the Discussion section, and the limitations of the dataset we used in this research. Finally, we summarize the proposed method in Conclusion section. 

## Materials and methods

### Model formulation

#### Survey response model

To analyze the grouped data generated by the questionnaire, we defined a mathematical model which described the process of answering grouped data type scales. The purpose of the questionnaire was to get the answer in participants’ minds. Within a population, the use of the random variable *X* denotes the answer in the mind of individuals. A *d* point scale *Q* maps an individual’s answer to a number in set $S=\{s_1, ..., s_d|s_i=i, i\in N\}$, and each element in set *S* has a corresponding numerical interval in set *I*. Assume that the union between these intervals in set *I* are always an empty set, we defined $I=\{[lb_i, ub_i)|i=1, ..., d\}$. Let *x* denote the answer of a respondent, and $x\in [lb_i, ub_i)$, then *Q* will map *x* to $s_i=i$. The data after mapping is called “grouped data”. Suppose that there are *n* independent respondents sample from the population and they all answer the questionnaire. We defined a vector ${\boldsymbol{o}}=[(o)_1, ...,(o)_d]^T$ which denotes the observation of each group. Furthermore, let probability vector ${\boldsymbol{p}}={\boldsymbol{o}}/n=[(p)_1, ..., (p)_d]^T$ denotes the relative frequency of each group, and it can also be seen as a discrete probability distribution of the survey responses. When it comes to measuring similarities between two discrete probability distributions, the first method that comes to our mind is L infinity. L infinity computes the maximum difference between each cell in different distributions, thus given two probability vectors *p* and *q* with the same dimension, L infinity between them is $L_\infty =max(|p_1-q_1|, |p_2-q_2|,...,|p_d-q_d|)$

Suppose that there are samples from the same population at different times, the probability vectors $p_1, p_2, ..., p_n$ denote the responses made by those samples at different times from $t_1$ to $t_n$. It is reasonable to denote the probability transitions between two probability vectors by a transition matrix. Let a transition matrix $T_1$ satisfies the equation $T_1*p_1=p_2$, a cell $(T_1)_{ij}$ in $T_1$ denotes the probability that an individual change their choice from option *j* at $t_1$ to *i* at $t_2$. Obviously, given $p_1$ and $p_2$ can’t solve the unique solution of $T_1$. But when some individuals appear multiple times (especially more than 2 times), say $t_1$ and $t_2$, then it is possible to use these individual’s responses to estimate $T_1$. We called these individuals “cohort” and defined cohort as the samples who have observations at two consecutive times. The transition matrix is constructed by multiple probability vectors, which can infer that measuring the distance between the transition matrices is similar to measuring the distance between pairs of probability vectors. Therefore, we defined the L infinity between two matrices as the maximum L infinity between every pair of probability vectors in those two transition matrices.

#### Long-term survey responses as multiple stochastic processes

We used stochastic processes to model responses to grouped data type scales at multiple times. According to the Survey Response Model defined earlier, the actual answer in the respondent’s mind is a real number. Consider a discrete time series $t_1,t_2,...$ , and a random variable *T* represents a specific time. Given a population that exists in all the times and the actual answer of individuals follows a distribution which changes depending on the time *T*. We used $X_T$ to denote the actual answer of individuals at *T*, then a discrete-time continuous state stochastic process $\{X_T\}_{T=t_i}$ can be constructed and the sample space of it is all of the real numbers. Suppose that we ask individuals to answer a grouped data type scale *Q* which maps answer in mind to a response option set *S* at every *T*, then we can get another stochastic process $\{Y_T\}_{T=t_i}$ with sample space *S* which denotes the survey responses at each time. Since grouped data type scales are one type of close-ended questionnaires, the mapping from $X_T$ to $Y_T$ can induce information loss and response bias. Hence, estimation of $X_T$ using observations of $Y_T$ can introduce systematic errors. In the Survey Response Model we defined earlier, ideally, the response bias does not occur, so the simulation experiment in this study, we only considered the systematic error induced by the information loss.

Considering that there are many versions of a questionnaire, then we can use distinct stochastic processes to denote the responses of each version of the questionnaire. Additionally, questionnaire revisions can be represented by the transitions between different stochastic processes. Different versions of a questionnaire cause different types of information loss and induce different systematic errors in the estimated answers in mind. Thus, to reduce the inconsistencies between estimations by observations from different questionnaire versions, transferring every observation from different stochastic processes to the same stochastic process before estimating the answer in mind is reasonable.

There are two perceptions of deciding the final transferred questionnaire version. Assume that a questionnaire with less information loss is likely to introduce fewer estimation errors, transferring to the questionnaire version with the least information loss is reasonable. On the other hand, since transferring the questionnaire version can cause some errors, transferring to the version that is used most of the time can reduce estimation errors.

When it comes to transferring observations from one stochastic process to another, we did not want the transitions that depended on time *T*. Thus, we proposed VAM, a method which can extract transitions depending on different interfering source factors separately. It is common to use only one version of a questionnaire to collect data at a single time. Hence, there are two types of transitions between the observations before and after revision; transitions according to time and transitions according to revision. These transitions can be denoted by conditional probability and can be composed to a transition matrix.

As mentioned previously, consecutive survey responses to the same questionnaire can be denoted by probability vectors. These probability vectors are the observations of a stochastic process $\{Y_T\}_{T=t_i}$, and the transition matrices can be used to represent the transitions that occur between the two consecutive times. If there is a questionnaire revision between $t_1$ and $t_2$, then the observations of $t_1$ and $t_2$ belong to different stochastic processes. To change the questionnaire version of observations in $t_1$ to $t_2$, we proposed that the first thing is to estimate the transition matrix which consists of time-related transitions and revision-related transitions, then decompose it into two transition matrices, one related to time and the other related to revision. After decomposition, the revision-related matrix can be used to change the questionnaire version of observations in $t_1$ to $t_2$.

It is difficult to estimate the transition matrix between $t_1$ and $t_2$ just by the observations in such times. A group of individuals that have observations in both $t_1$ and $t_2$ is labelled as cohort. The observations of the cohort can be used to estimate the transition matrix directly. It belongs to a stochastic process that only has observations two consecutive times and is different from the stochastic process of the whole population. By using the method we proposed, the cohort revision-related matrix can be estimated. By assuming that the reaction of revision in different groups of individuals is the same, the cohort revision matrix is identical to the population revision matrix. Finally, the population revision matrix can be used to change the version of the questionnaire used to collect observations in $t_1$ to the version of the questionnaire used in $t_2$.

### Algorithm VAM

The main purpose of VAM is to reduce the inconsistency between the grouped data collected from two different versions of a questionnaire. These grouped data were collected at multiple times, and the version of questionnaire is different each time. Because of the change of the version, using the responses from different times to estimate the answer in the respondents’ mind can introduce different systematic errors and cause inconsistent estimations.

**Fig. 1:**
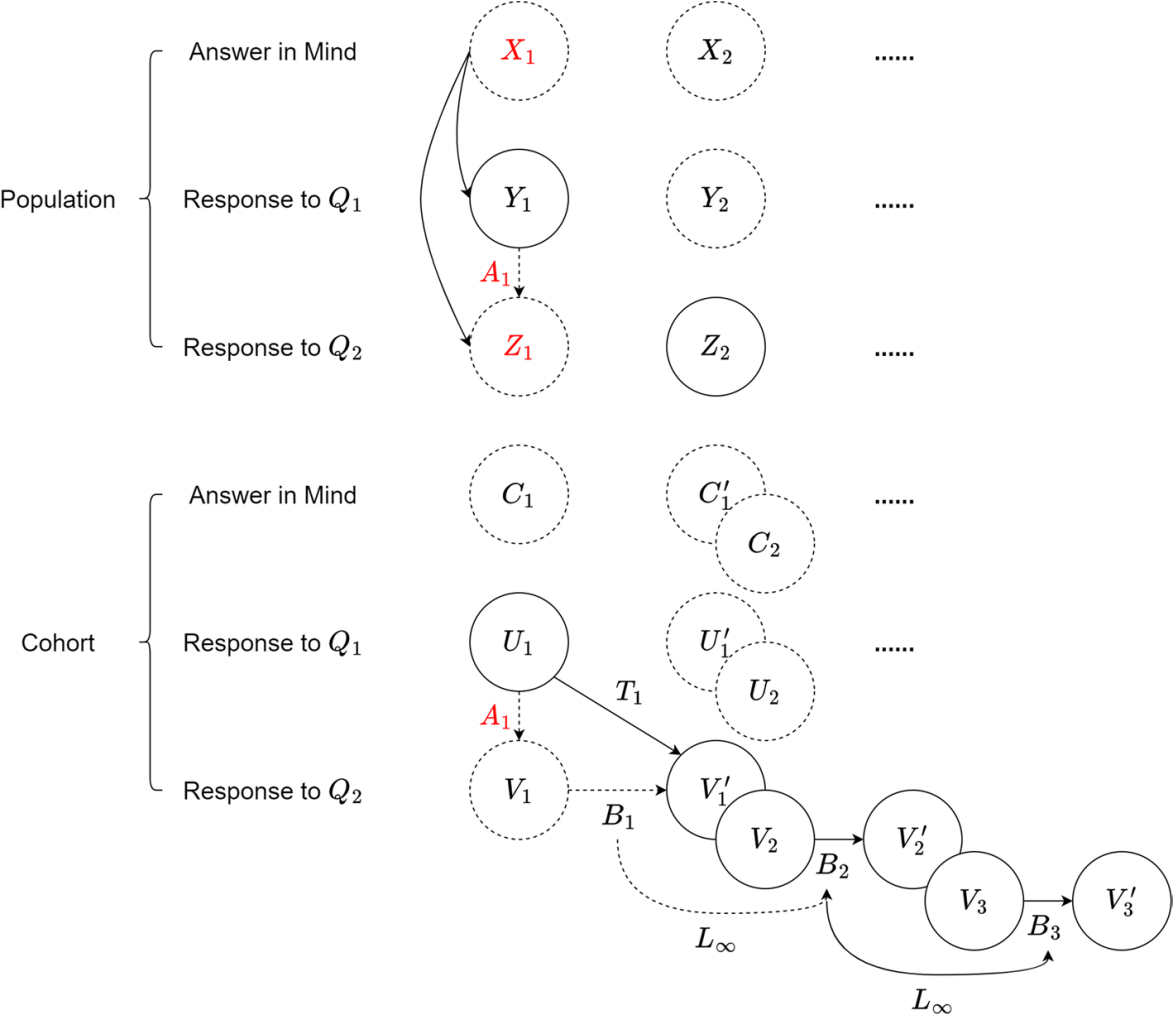
Scenario of inputs of VAM. The non-dashed lines and circles represent given information. And the letters in red represent the estimation target

The inputs of VAM are time-series data with multiple stochastic processes, and the scenario of it is depicted in Fig. 1. Given a stochastic process $\{X_i\}$, which represents the population’s answer in mind and follow the unknown distribution $F(\theta _i)$ at each time $t_i$. As VAM aligns observations from two different versions of a questionnaire, assume only two versions of a questionnaire, $Q_1$ and $Q_2$, are used to collect responses from the population and the responses can form two additional discrete state stochastic processes $\{Y_i\}$ and $\{Z_i\}$. In addition, a group of individuals who are observed by questionnaire in multiple consecutive times is called a cohort $\{C_i\}$ with unknown underlying distribution. The responses of cohort form another two stochastic processes’ sets $\{\{U_i, U'_i\}\}$ and $\{\{V_i,V'_i \}\}$, and corresponding to $Q_1$ and $Q_2$ respectively. Using the observations of cohort, the cohort transition matrix $T_i$ between two consecutive times can be easily estimated, while the transition matrix of the population can not be estimated easily. As the cohort and population are not from the same stochastic process, their transitions are different even if they occur at the same time, which implies their transition matrices are different. Thus, VAM leverages the transition matrix of cohorts to estimate the revision-related matrix, and then adopts the revision-related matrix for reducing the revision effect that occurs in population.

Assume that a revision happened between $t_1$ and $t_2$, and the version of the questionnaire is changed from $Q_1$ to $Q_2$. Then we can only observe responses of $Y_1$, $Z_2$, $U_1$, $V'_1$, and $V_2$ in $t_1$ and $t_2$. When we attempt to estimate the expected value of $X_1$ and $X_2$ by observations of $Y_1$ and $Z_2$, the estimated expected values $\hat{E}(F(\theta _1))$ and $\hat{E}(F(\theta _2))$ will include different systematic error and cause inconsistency, since $Y_1$ and $Z_2$ came from different stochastic processes corresponding to different versions of the questionnaire with different information loss and response bias. Suppose that the information loss of $Q_2$ is less than $Q_1$, and $Q_2$ is used more than $Q_1$. It is reasonable to map all the observations of $\{\{U_i, U'_i\}\}$ and $\{Y_i\}$ corresponding to $Q_1$ to $\{\{V_i, V'_i\}\}$ and $\{Z_i\}$, because the estimation of expected value has a larger probability to include less estimation error if the information loss and the estimation error of switching between different stochastic processes decreases. The transitions of $\{\{V_i, V'_i\}\}$ after $t_2$ are only related to change of time, and so are the transitions of $\{\{U_i, U'_i\}\}$ before $t_0$. Note that there are two kinds of transitions observed at $t_1$, which are revision-related transitions and time-related transitions, and these transitions compose transition matrix $T_1$. The time-related transitions can compose a transition matrix, $B_1$, and the revision-related transitions can compose a transition matrix, $A_1$. It is intuitive to form the equation of the cohort transitions in $t_1$ as

1 $$\begin{aligned} T_1*u_1=B_1*A_1*u_1=B_1*v_1=v'_1 . \end{aligned}$$

$u_1,v_1,v'_1$ represents the discrete distribution of the random variable $U_1,V_1,V'_1$, respectively. The Eq. (1) reveals that observations of $U_1$ can be transferred to $V'_1$ by the transitions of $T_1$ or by combination of transitions of $B_1$ and $A_1$. Except $t_1$, there is only time-related transitions in other times; hence, the revision-related transitions can be formed as a identity matrix *I*, which do not contain any effective transition. As the goal is to reduce the systematic error inconsistency, which is induced by revision, by transferring the observations from $Y_1$ to $Z_1$, we need to extract the revision-related transition matrix $A_1$ from $T_1$. Assume that the revision effect is the same in every group of individuals, thus revision-related transitions are identical in population and the cohort. Thus, $Y_1$ and $Z_1$ are from population stochastic processes but the revision-related transitions between them are equal to the transitions between $U_1$ and $V_1$, which compose transition matrix $A_1$. Finally, multiplying $Y_1$ by $A_1$ gives $Z_1$, which is the estimated responses to $Q_2$ of the population at $t_1$.

After the application of the revision-related transition matrix $A_1$ to $Y_1$ gives the estimate grouped data after revision, $Z_1$, using it can estimate the proper $\hat{E}(F(\theta _1))$, which contains the systematic error corresponding to $Q_2$. This reduces the inconsistency caused by a different systematic error, because $\hat{E}(F(\theta _i))|_{i\ge 2}$ contains the systematic error corresponding to $Q_2$. Moreover, $A_1$ is estimated from cohort probability vector (p.v.) and transition matrices, and it is composed of cohort revision-related transitions; therefore it can be truly applied to $U_1$. Thus, the output of VAM includes $Z_1$, $V_1$, the matrices $B_1$ and $A_1$, and the estimation of the mean of population, $\hat{E}(F(\theta _1))$.

It is impossible to use Eq. (1) to solve the $B_1$ and $A_1$. Therefore, we introduced six constraints, which describes the similarity between vectors and matrices, to construct an optimization problem that makes $B_1$ and $A_1$ solvable. Four of the six constraints are listed below.

$$\mathbf {C_1}$$
The columns in transition matrix must sum to 1, then by assuming $B_1$ is an n by n matrix, the first constraint is denoted by $\sum _{k=1}^{n}(B_1)_{kj}=1, j=1,...,n$. The $(B_1)_{kj}$ denotes the entry at *k*th row and *j*th column of $B_1$.
$$\mathbf {C_2}$$
$A_1$ is also an transition matrix, then by assuming it an n by m matrix, the second constraint is denoted by $\sum _{k=1}^{n}(A_1)_{kj}=1, j=1,...,m$. The $(A_1)_{kj}$ denotes the entry at *k*th row and *j*th column of $A_1$.
$$\mathbf {C_3}$$
According to Eq. (1), constraint equation $B_1*A_1=T_1$ can be realized. As the estimation of $A_1$ and $B_1$ introduces some tolerable but inevitable error and the precision of decimal in probability is set to the fourth decimal places, it is reasonable to change the equality to approximate equality. Due to the distance measurement method we used is L infinity, constraint can be rewritten as $|B_1*A_1-T_1|_\infty <\gamma$. $\gamma$ represent the error that can be tolerated.
$$\mathbf {C_4}$$
According to Eq. (1), constraint equation $B_1*A_1*u_1=v'_1$ can be realized. As the same reason mentioned in $\mathbf {C_3}$, constraint can be rewritten as $|B_1*A_1*u_1-v'_1|_\infty <\epsilon$. $\epsilon$ represent the error that can be tolerated.

In addition to the four constraints introduced, the remaining two constraints are according to the assumptions on time-series data and the underlying distribution.

$$\mathbf {C_5}$$
We assume two time-related cohort transitions in consecutive times are similar, because we believed that the individuals in the cohort read the health screening report and react in similar ways, as well as that the trend of LTPA duration is similar in consecutive times. Hence, the corresponding transition matrices, $B_1$ and $B_2$, are similar and the constraint can be formulated as $|B_1-B_2|_\infty <\beta$. $\beta$ denotes the similarity of $B_1$ and $B_2$.
$$\mathbf {C_6}$$
According to the proposed Survey Response Model and assumption of the underlying distribution of cohort in $t_1$, we can manually compute an ideal revision matrix that consists of ideal transitions, which represent the change of individuals’ responses of $Q_1$ to responses of $Q_2$ at $t_1$. We assumed this ideal transition matrix is similar to the revision matrix, $A_1$, which we aim to estimate. Assume that the PDF of the underlying distribution of cohort at $t_1$ is $H(\xi _1)$, and by using MLE to the observations of $U_1$ ,the $\hat{\xi }_1$ can be estimated. Suppose that the interval set $I_1=\{(I_1)_j=[lb_j, ub_j)|j=1, 2, ..., d_1\}$ belongs to $Q_1$, and $I_2=\{(I_2)_i=[lb_i, ub_i)|i=1, 2, ..., d_2\}$ belongs to $Q_2$, then an ideal transition matrix of revision can be computed, denoted by *G*. $d_1$ and $d_2$ denote the number of response options for $Q_1$ and $Q_2$. Define a function $\Phi (interval)$, which denotes the cumulative probability of $H(\hat{\xi }_1)$ in a given interval. Then each cell in *G* can be computed by the following equation:
  $$\begin{aligned} G_{ij}= & {} p(x\in (I_2)_i|x\in (I_1)_j)\\ = & {} p(x\in (I_1)_j\cap x\in (I_2)_i)/p(x\in (I_1)_j)\\ = & {} \Phi ((I_2)_i\cap (I_1)_j)/\Phi ((I_1)_j), \end{aligned}$$
  and it describes the probability of changing from $Q_1$’s response option *j* to $Q_2$’s response option *i*, and *x* in the equation denotes the answer in the respondent’s mind. According to the assumption, which is the ideal revision-related transition matrix *G* will be similar to the real revision matrix $A_1$. Then the last constraint $|A_1-G_1|_\infty <\alpha$ can be constructed.

The six constraints each represent a different relationship between vectors or matrices, and some of them use variables of similarity$(\epsilon , \gamma , \beta , \alpha )$ to denote the similarity between vectors or matrices in each relationship. Moreover, except for the two constraints related to transition matrix’s basic property that does not include a variable of similarity, which has no tolerance on that the probabilities in a column of a transition matrix need to be sum to 1. The variable of similarity in other constraints have a proportional relationship between each other. The proportional relationship represents the order of similarity relevant to other constraints, the higher proportion implies the higher similarity of vectors or matrices in that constraint. Let $\theta _i$ denotes the proportion between those variables of similarity and we named it the parameter of proportion. $\theta _1$ to $\theta _4$ are according to variables of similarity $\gamma$, $\epsilon$, $\beta$, and $\alpha$ respectively. As the variable of similarity is the distance in L infinity, and according to the rationale of constraints that the vectors or matrices are similar and have subtle distance between them, the objective function can be defined as a minimization equation as follows.

2 $$\begin{aligned} min\ \theta _1*\gamma +\theta _2*\epsilon +\theta _3*\beta +\theta _4*\alpha \end{aligned}$$

subject to

3 $$\begin{aligned} \sum\limits_{k=1}^{n}(B)_{kj}=1, j=1,...,n \end{aligned}$$

4 $$\begin{aligned} \sum\limits_{k=1}^{n}(A)_{kj}=1, j=1,...,m \end{aligned}$$

5 $$\begin{aligned} |B_1*A_1-T_1|_\infty < \gamma \end{aligned}$$

6 $$\begin{aligned} |B_1*A_1*U_1-V'_1|_\infty <\epsilon \end{aligned}$$

7 $$\begin{aligned} |B_1-B_2|_\infty <\beta \end{aligned}$$

8 $$\begin{aligned} |A_1-G_1|_\infty <\alpha \end{aligned}$$

This is a Quadratic Programming (QP) problem. The proportional relationship can be determined by comparing the rationale of each constraint. The inequality constraints (5) and (6) according to the Eq. (1), which can only tolerate the subtle estimation error below $10^{-4}$ from estimating probability vectors (e.g. $u_1,v'_1$) and transition matrices (e.g. $A_1,B_1$), and also tolerate the precision of probability to $10^{-4}$. It implies that we need to set the highest priority of minimizing the corresponding variables of similarity and below $10^{-4}$. Accordingly, the corresponding $\theta _1$ and $\theta _2$ are equal to each other and are greater than other parameters of proportion. After that, the remaining two inequality constraints (7) and (8) are corresponding to assumptions of time-series data and the Survey Response Model with an known underlying distribution. As we had more confidence in assuming transition matrix is alike at consecutive times, the similarity in constraint (7) is higher and has a greater relevant proportion $\theta _3$. Furthermore, we assumed that the distance of the transition matrices is alike when the matrices are consecutive and the duration of transitions of the matrices are similar. Thus, the distance between $B_1$ and $B_2$ may be close to the distance between $B_2$ and $B_3$, which is a reasonable reference to the variable of similarity $\beta$ when tuning the parameter of proportion during the optimization process. The last constraint (8) is the similarity between the ideal revision-related matrix *G* and the actual revision-related matrix $A_1$, which has the least priority on minimization because the distribution assumption is arbitrary and the ideal revision-related transitions may vary from the realistic revision-related transitions. In summary, the parameter of proportion acts like hyperparameter which is tuned in the optimization process but needs to satisfy the inequality $\theta _1=\theta _2>\theta _3>\theta _4$, and make the corresponding variable of similarity meet the target value in the end.

### MJ dataset

The relationship between leisure-time physical activity (LTPA) and various diseases has been well studied via analyzing various datasets. Since the MJ dataset collected a large amount of long-term medical screening data, many researchers have used it as evidence to support their argument [8, 9]. MJ dataset includes demographics, lifestyle, and medical history data collected by a self-administered questionnaire. Additionally, a series of health examinations including anthropometric measurements, general physical examinations, and biochemical tests of blood and urine were taken, which complements the corresponding data [10]. Most of this study used the intensity of LTPA and duration spent on LTPA in the MJ dataset to compute the level of LTPA. The questionnaire asking the duration of LTPA is a grouped data type scale, and it was revised several times during data collection.

**Fig. 2:**
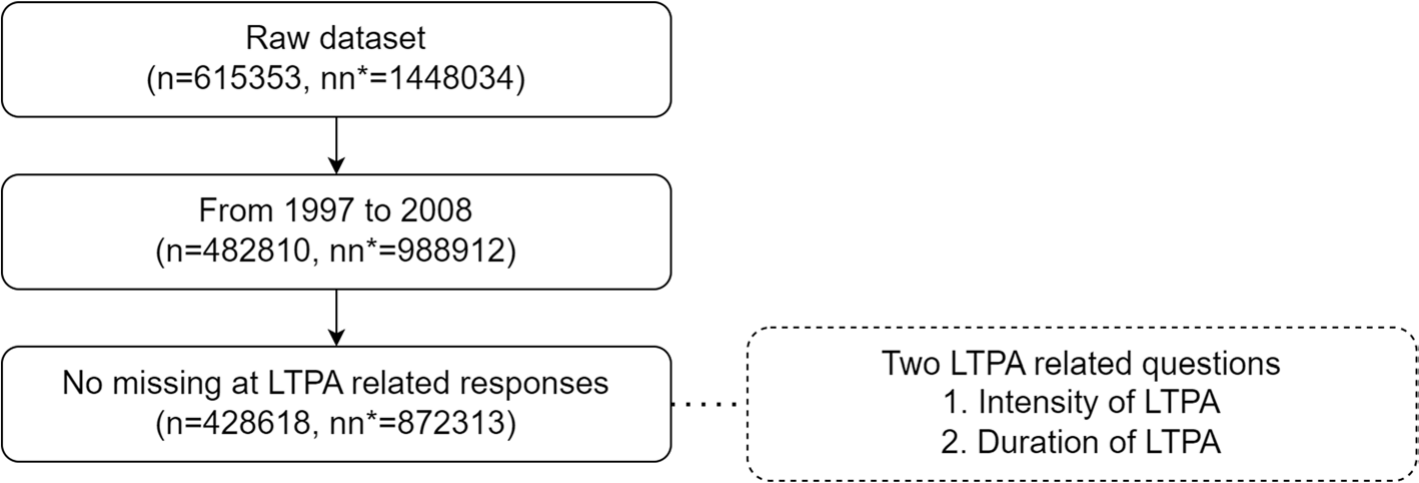
Process of screening the MJ dataset. n denotes the number of individuals. nn denotes the number of observations, including multiple re-screenings

There were 615,353 individuals (1448034 responses including multiple screening records) who participated in the health screening program run by MJ Health Management Institution, and all of their screening data were between 1996 and 2017. Due to the purpose of this study, which is to remove the effects caused by revisions, we used the data from 1997 to 2008 that contained a version changed on the questionnaire asking for the duration of LTPA. Since the level of LTPA (MET-h per week) was computed by LTPA intensity (MET; 1 MET=1 kcal per h per kg of bodyweight) times LTPA duration (hour/week)[8], only the participants who responded both intensity and duration were considered. Besides selecting data in specific years and responses to specific questions, due to how the population cannot estimate the transition matrix directly, we additionally selected the individuals who rescreened in two consecutive years to estimate the transition matrices directly. These individuals are defined as the cohorts. Fig. 2 describes the process of data screening.

There are several versions of the multi-choice questionnaire asking the duration of LTPA. They all share the same statement *Fixed duration for exercise* from 1997 to 2008, but have different descriptions of response options. The first version only contained four options, (1) *No or less than one hour a week* (2) *One to two hours a week* (3) *Two to three hours a week* (4) *Above 3 hours a week*, this version was only used in 1997, and we named it “questionnaire of 1997”. Additionally, this version is denoted by $Q_{1997}$, and the response to this version at multiple times can be described by a stochastic process. It’s sample space is $S=\{1, 2, 3, 4\}$ and the corresponding interval set is $I=\{[0, 1), [1, 2), [2, 3), [3, \infty )\}$. The second version was used between 1998 to 2008 and contained 5 options, (1) *No or less than 1 hour a week* (2) *1 to 2 hours a week* (3) *3 to 4 hours a week* (4) *5 to 6 hour a week* (5) *More than 7 hours a week*, we call this version “questionnaire of 1998”. Obviously, the numerical interval of options in the questionnaire from 1998 is discontinuous, thus assuming that the respondent answered the interval closest to the answer in their mind when the answer was not in any interval of the options. The discontinuous gap was evenly distributed to adjacent intervals. For example, the upper bound of option 2 was extended to 2.5 hours, and the lower bound of option 3 was extended to 2.5 hours. Same as the $Q_{1997}$, we denoted questionnaire of 1998 as $Q_{1998}$ and the responses is described by a stochastic process different from $Q_{1997}$. The sample space of it is $S=\{1, 2, 3, 4, 5\}$ and the corresponding interval set is $I=\{[0, 1), [1, 2.5), [2.5, 4.5), [4.5, 6.5), [6.5, \infty )\}$.

**Fig. 3:**
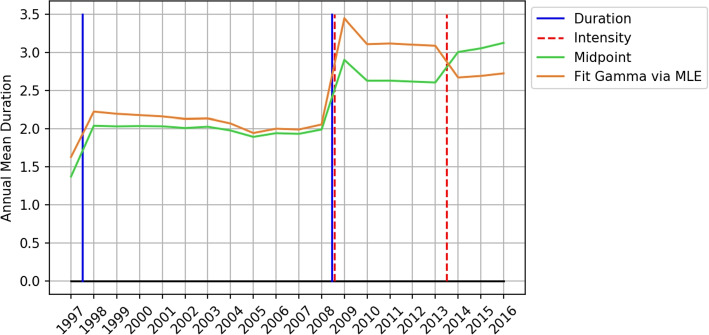
Yearly mean of weekly duration of LTPA. The vertical lines in the figure show the revision time of duration and intensity questions, which are the blue and the dotted red lines, respectively. The orange line shows the annual mean duration, estimated by the midpoint method. The green line shows the annual mean duration estimated by MLE

**Fig. 4:**
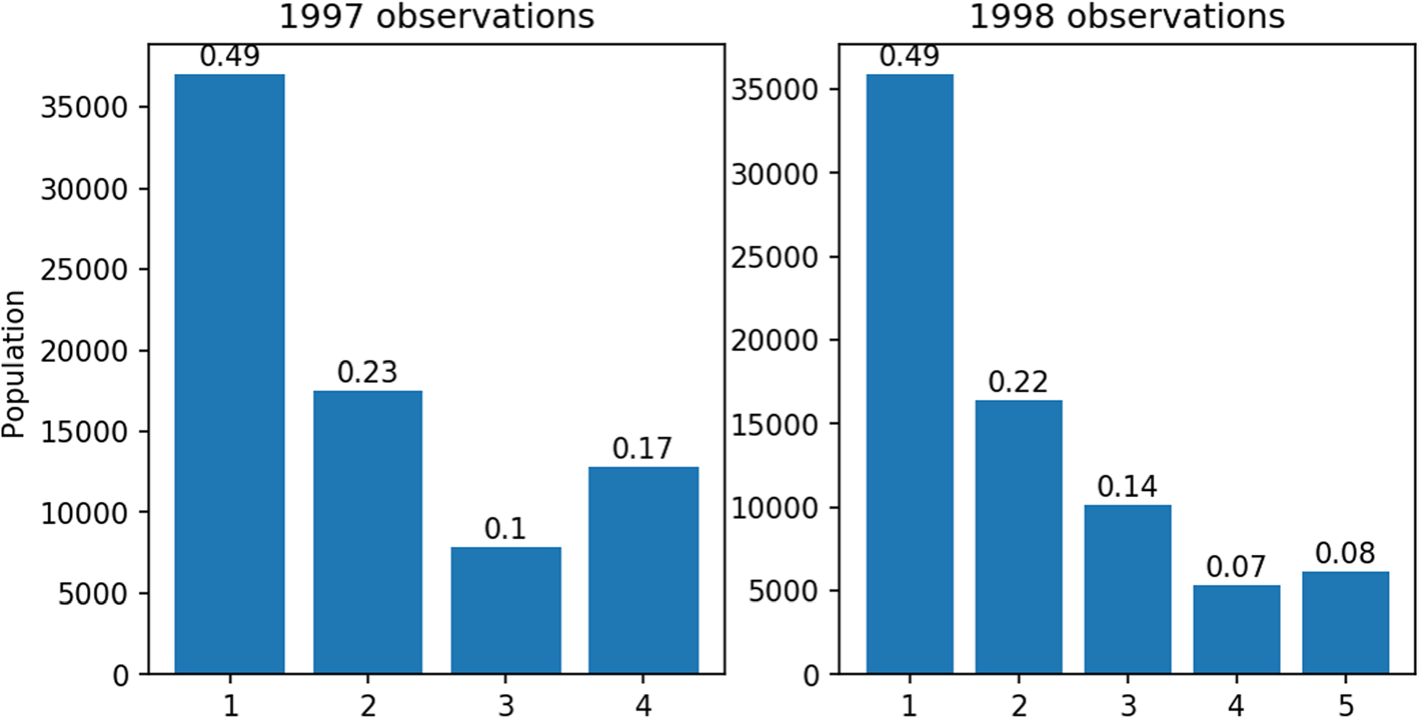
Distribution of grouped data in 1997 and 1998. The left figure shows the distribution of survey responses in 1997, and the right figure shows the distribution of survey responses in 1998. The height of each bar is the number of observations of that response in a specific year. The number above each bar is the relative frequency of that response

As stated earlier, the questionnaire of duration is a grouped data type scale and the data collected is grouped data. Most of the studies were interested in the annual average physical activity change, since the level of LTPA is LTPA intensity times LTPA duration, and it is necessary to compute the annual average duration of LTPA first. To our best knowledge, no study had reported their strategy on analyzing the average duration of LTPA and the strategy on dealing with revision, hence the method that computes average LTPA remains unclear. Therefore, researchers that want to study similar topics will need to devise a method on their own, which can lead to unexpected or various results. When it comes to computing the average of grouped data, the most simple way is to use the midpoint of the interval corresponding to each option as the average of that option. This assumes that responses in each interval are uniformly distributed, and we named this naive method the midpoint method. The mean of the last option with an interval that has an infinite upper bound is unable to be estimated properly, even assuming the observation is uniformly distributed can still get 0 mean. It is intuitive to set the lower bound of that interval as the mean of that interval, but this can easily underestimate the mean of that option. According to the description of the midpoint method, the midpoint of each options in $Q_{1997}$ are $\{0.5, 1.5, 2.5, 3\}$. Assume that the probability distribution of observation of each option is $[p_1, p_2, p_3, p_4]$, then the annual average duration of 1997 by the midpoint method is $0.5*p_1+1.5*p_2+2.5*p_3+3*p_4$. We used the midpoint method to compute the average of LTPA duration in the MJ dataset and found a large gap between the duration of LTPA in 1997 and 1998, which is shown in Fig. 3 by the green line. Due to the problem caused by the option with an infinite upper bound, both LTPA duration in 1997 and 1998 are underestimated. Moreover, the distribution of responses in Fig. 4 shows that the relative frequency of the last response option is larger in 1997, which implies that the underestimation in 1997 is larger, which also explains the large gap between LTPA duration in 1997 and 1998. The higher underestimation in 1997 can be explained by the higher information loss in $Q_{1997}$. $Q_{1998}$ preserves some information on the distribution greater than 3 which is completely missing in $Q_{1997}$. Besides that, $Q_{1997}$ was only used in 1997 but $Q_{1998}$ was used from 1998 to 2008. It is reasonable to transform to the version that is used most of the time which can reduce the number of transformations between stochastic processes, and reduce the estimation error introduced by the transformations. In summary, transforming all the observations of the stochastic process related to $Q_{1997}$ to the stochastic process related to $Q_{1998}$ is more feasible. Thus, in this study, we transformed the observations in 1997 to the stochastic process related to $Q_{1998}$.

The evidence mentioned above shows that the midpoint method isn’t feasible to use when there is an infinite bound appearing in any interval. Additionally, it is not appropriate to consider the underlying distribution as multiple uniform distribution segments, which is dissimilar from the distribution of actual responses. Xiao, X. et al. [5] proposed that MLE is the most robust method to analyze grouped data when not enough prior information is available. MLE takes the infinite upper bound into account and considers the underlying distribution as a proper continuous distribution, which makes it more reliable than the midpoint method. Using MLE to estimate expected value is as simple as the midpoint method, and an example of estimating expected value of the annual average LTPA duration of 1997 is shown below. Suppose that the underlying answer in the respondents mind follows a Gamma Cumulative Distribution Function (CDF) which is denoted by $F(ub_i, \theta )$, where $ub_i$ denotes a upper bound which related to a option of $Q_{1997}$, and $ub_0=0$. The parameter $\theta$ of the CDF denotes the parameter of Gamma distribution. Furthermore, we denoted the observed frequency of response options of $Q_{1997}$ by ${\varvec{o}}=[(o)_1, (o)_2, (o)_3, (o)_4]$. It follows the probability mass function $g((o)_1, (o)_2, (o)_3, (o)_4;\theta )=\frac{((o)_1+(o)_2+(o)_3+(o)_4)!}{(o)_1!(o)_2!(o)_3!(o)_4!}\prod _{i=1}^{4}[F(ub_i,\theta )-F(ub_{i-1},\theta )]^{(o)_i}$. The best $\theta$ fitting $g((o)_1, (o)_2, (o)_3, (o)_4; \theta )$ can be solved by MLE and the expected value of annual average LTPA duration of 1997 can be estimated by $\theta$. The orange line in Fig. 3 is the annual average duration of LTPA estimated by MLE when assuming the underlying distribution is Gamma distribution. The average estimated by MLE is larger than by the midpoint method because the infinite upper bound is taken into account. But the large gap between 1997 and 1998 still exists, even larger, hence the effect caused by revisioning the questionnaire is huge and cannot be corrected by MLE. The estimated annual means computed by the midpoint method and MLE are listed in Table 1 column 3. There is a larger difference in the annual average duration of LTPA between 2008 and 2009 shown in Fig. 3. During the same time, the item asking LTPA duration was revised into two questions belong to two items, and the item asking LTPA intensity was revised. This made the revision effect more complicated, we did not use data after 2008 in this study. The item asking LTPA intensity was revised again between 2013 and 2014. After this revision, respondent is able to skip items asking LTPA duration according to their response to item asking LTPA intensity. As the branching logic was introduced into the questionnaire, a significantly change on annual mean of LTPA duration occurred between 2013 and 2014. Additionally, there were multiple items asking LTPA duration and LTPA intensity revised between 2016 and 2017, and we did not use observations in 2017 as well. The questionnaire used in 1996 is not a grouped data type scale, since the response options did not contain actual numerical intervals. We did not use observations in 1996.

**Table 1:** Information of all observations. This table lists the statistics of the population in each year. The number of individuals is listed in the *Population* column. The estimated annual mean of LTPA duration of the population is listed in the *Mean(Midpoint, MLE(Gamma))* column. The absolute difference of annual mean in two consecutive years are listed in the *Diff(Midpoint, MLE(Gamma))* column. The L infinity between probability vectors of population consecutive years is listed in the *L infinity between vectors* column. * Due to the revision between 1997 and 1998, the Linfinity of probability vectors can’t be computed. All the probabilities are rounded to 5 decimal places

Year	Population	Mean(Midpoint, MLE(Gamma))	Diff(Midpoint, MLE(Gamma))		L infinity between vectors	
1997	75242	1.36833, 1.62673	0.68243, 0.59378		*X	
1998	73940	2.05077, 2.22051		0.00320, 0.02901		0.01263
1999	70316	2.04756, 2.19150	0.00896, 0.01719		0.01135	
2000	74962	2.05653, 2.17430		0.00038, 0.01712		0.00941
2001	67811	2.05691, 2.15717	0.02025, 0.03129		0.00395	
2002	69715	2.03665, 2.12588		0.02099, 0.00590		0.01431
2003	63852	2.05765, 2.13179	0.04719, 0.06497		0.00875	
2004	71137	2.01045, 2.06681		0.07386, 0.12550		0.01437
2005	74616	1.93659, 1.94131	0.04867, 0.05672		0.01634	
2006	77411	1.98527, 1.99804		0.00836, 0.01061		0.00266
2007	76808	1.97690, 1.98742	0.05552, 0.06614		0.01216	
2008	76503	2.03243, 2.05356				

The difference in annual means from 1998 to 2008 was listed in the fourth column in Table 1. And the range of it is [0.00038, 0.07386] and [0.00590, 0.12550], computed by the midpoint method and MLE, respectively. Besides that, the difference of the estimated annual mean between 1997 and 1998 is 0.68243 by midpoint method and 0.59378 by MLE. Additionally, it is increasing between 1997 and 1998 while decreasing between 1998 and 2008. The difference of annual mean between two consecutive years can represent a short-term LTPA change, and we assume that the amount of short-term LTPA change did not vary extremely. However, the difference between 1997 and 1998 was not close to the annual mean ranges from 1998 to 2008. Since there was no other event that happened in Taiwan between 1997 and 1998 which affected people’s habits of physical activity during leisure time, it is reasonable to deduct questionnaire revisions as the source of interference.

The data collected by the questionnaire of duration is grouped data. Additionally, we considered it as a probability vector or a discrete probability distribution in one year and computed the L infinity between two consecutive years. The 5th column in Table 1 listed the L infinity from 1998 to 2007, it can be found that the maximum L infinity is 0.0163 indicates the maximum difference of relative frequency between two consecutive years is $1.6\%$ which is a small value. As we assumed that short-term LTPA change is subtle, the L infinity between 1997 and 1998’s probability vectors must be close to the L infinity between 1998 and 1999’s probability vectors or $1.6\%$.

In addition to the probability vector computed by all the observations, some special groups of individuals who were rescreened in two consecutive years can also use the same method to compute multiple pairs of probability vectors. These groups of individuals are defined as cohorts. The L infinity of cohorts from 1998 to 2007 are all greater than the L infinity computed by all the observations. We simply deduced the reason was the behavior changed caused by having access to and reading the health screening reports. The L infinity of cohorts are listed in the 3rd column in Table 2. Since it is possible to estimate the transition matrices of cohorts directly, the cohort transition matrices were estimated and the L infinity between them was listed in Table 2, column 4. Note that the transition matrix of 1997 and 1998 includes revision transitions, thus it is not compatible with other transition matrices and have different dimensions with them. In this study, we estimated the transition matrix of 1997 and 1998 without revision transitions, and we assumed that the L infinity between it and the transition matrix of 1998 was close to the L infinity between the transition matrix of 1998 and the transition matrix of 1999.

**Table 2:** Information of the cohort. This table lists the statistics of the cohort in each two consecutive years. The number of cohort individuals is listed in the *Rescreen population* column. The L infinity between the two consecutive cohort transition matrices is listed in the *L infinity norm between transition matrices* column. The L infinity between the two consecutive cohort probability vectors is listed in the *L infinity norm between vectors* column. * Due to the revision between 1997 and 1998, the L infinity of probability vectors and transition matrices can’t be computed. All the probabilities are rounded to 5 decimal places

Year	Rescreen population	Linfinity between vectors	Linfinity between matrices	
1997-1998	21551	*X	*X	X
1998-1999	20461	0.04183		0.03200
1999-2000	22245	0.03735	0.04007	
2000-2001	21914	0.04303		0.02244
2001-2002	20257	0.02749	0.04503	
2002-2003	19577	0.04178		0.04052
2003-2004	21186	0.01444	0.05387	
2004-2005	23928	0.01830		0.05048
2005-2006	24903	0.03919	0.02865	
2006-2007	25648	0.02144		0.03269
2007-2008	27018	0.03664	X	

It is possible to estimate the transition matrix by cohort. Table 2 column 4 lists the L infinity between transition matrices, and the range of L infinity between matrices is [0.022, 0.054]. We can infer that L infinity between 1997 and 1998 will be close to this range. In summary, L infinity between vectors and matrices, without the effect of revisions, may be useful when estimating the probability vector or the annual average at 1997.

### Simulation

As the underlying distribution is unknown in the MJ dataset, the actual error of the estimated result cannot be computed. To verify that the proposed method only corrected the error caused by revision and did not produce unexpected errors in the estimated underlying distribution, a dataset that can provide the underlying information of population was required. Simulation is a means to generate a controllable dataset. We constructed a simulation process by referring to the Survey Response Model defined earlier. In the simulation, the most important parts needs to be simulated are the pairs of cohort’s p.v. and the revision-related transitions and time related-transitions between them. This is because the proposed method tried to estimate the matrix composed of those transitions and apply the estimation to population’s p.v. for reducing the revision effect. The probability vectors and transition matrices in MJ dataset are utilized in the simulation process. This made the simulation result similar to that of the scenario in the real dataset. Sample size, questionnaire, and transition matrices in the MJ dataset were used as configurations in simulation. A general simulation process is considered, which includes some random factors in generating time-related transition matrices. The transition matrices in the MJ dataset between 1998 and 2008 are used for reference, since they only consists of time-related transitions. The diagonal elements in the generated transition matrix is computed by sampling a number from a uniform distribution. The lower and upper bound of the uniform distribution are the minimum and maximum of diagonal elements in the transition matrices refer to MJ dataset. The non-diagonal elements of the generated transition matrix is computed by weighted average of the non-diagonal elements in the transition matrices refer to MJ dataset. The weights used in weighted average are generated from uniform distribution with 0 as lower bound and 1 as upper bound. After weighted sum, the non-diagonal elements in each column is divided by the sum of non-diagonal elements in that column. Finally, multiply the non-diagonal elements and the difference of 1 and the diagonal element in the same column. There were three simulation datasets been generated with different transition matrices, but the other configurations remains the same.

According to our purpose of doing simulation, we need to simulate a transition that includes the effect of revision to a cohort which has observations in two different times. The simulation scenario is similar to the scenario in MJ dataset, which is depicted in Fig. 1, but only two times ($t_1$ and $t_2$) are considered. Given a population $X_1$ follows a underlying distribution with Probability Density Function(PDF) $F(\theta )$, which denotes the answer in the mind of the population at $t_1$. We used $F(\theta )$ to draw samples and used them to complete two versions of a questionnaire $Q_1$ and $Q_2$ where two random variables, $Y_1$ and $Z_1$, denote survey responses. Furthermore, $y_1$ and $z_1$ denote the corresponding discrete distribution of survey responses. $Q_1$ and $Q_2$ are the same as $Q_{1997}$ and $Q_{1998}$, which were used in the MJ dataset. Randomly selecting data from the samples just drawn from $F(\theta )$, gives the cohort random variables $U_1$ and $V_1$ denote the survey responses at $t_1$, and $u_1$ and $v_1$ denote the corresponding discrete distribution of the cohort’s survey responses. As noted earlier, we randomly generated some time-related transition matrices based on the transition matrices in the MJ dataset between 1998 and 2008. Those matrices were used as the cohort time-related transition matrix between $t_1$ and $t_2$ in simulation, denoted by $B_1$. The cohort time-related transition matrix between $t_2$ and $t_3$ in simulation used another generated transition matrix, denoted by $B_2$. The L infinity between $B_2$ and an imagine $B_3$ for reference was the average L infinity between all the pairs of the generated transition matrices. Therefore, we multiply $B_1$ by grouped data of $V_1$ gives $V'_1$ denotes the cohort completing questionnaire $Q_2$ at $t_2$. After that, the cohort transition matrix $T_1$ was constructed by transitions between $U_1$ and $V'_1$, since every individual’s ID and their responses at every time were known. After the procedure of generating a simulation dataset, we defined the estimation task similar to the scenario in the MJ dataset. Assume that we can only observe grouped data collected by $Q_1$ at $t_1$, and grouped data collected by $Q_2$ at $t_2$. Grouped data of all the observations $Y_1$, grouped data of cohort $U_1$, $V'_1$, the cohort transition matrix $T_1$, $B_2$, and the L infinity of $B_2$ and $B_3$ are given in the estimation task. The goal of the estimation task is estimating the response of cohort and of all the observations to $Q_2$ at $t_1$, $z_1$ and $v_1$, and the expected values of the underlying distribution of population at $t_1$, $\hat{E}(F(\theta ))$. Additionally, the estimation error needed to be computed to verify that the proposed method VAM is feasible. The configuration parameters were listed in Table 3.

**Table 3:** Configuration parameters in simulation. Since the underlying distribution and sample size in $t_2$ were not used in the simulation, they were not specified and denoted by an X. $v_1$ denotes the cohort completing questionnaire $Q_2$ at $t_1$. Both $B_1$ and $B_2$ were randomly generated based on the transition matrices in the MJ dataset between 1998 and 2008

Time	$t_1$	$t_2$
Underlying distribution of population	*Gamma*(1, 3)	X
Sample size	57000	X
Questionnaire version	1997	1998
Generation method of cohort grouped data	Randomly select from samples	Computed by $B_1*v_1$
Transition matrix $B_1$	Randomly generated	
Transition matrix $B_2$	Randomly generated	
Cohort size	20000	

To implement the VAM method and the simulation experiment, we used the Scipy [11] package in python for linear regression analysis, nloptr [12] package in R for Maximum likelihood estimation, and Optimization Toolbox add-on in Matlab [13] for solving quadratic programming problems. The code of estimation and simulation is available at github repository [14]. Part of the simulation data and estimations are available at Supplementary material.

## Result

### The MJ dataset

In the MJ dataset, a revision occured between 1997 and 1998 on the questionnaire investigating the LTPA duration, and we wanted to reduce the inconsistency that it induced. As the responses to the 1998 questionnaire had a smaller relative frequency on the group with an infinite upper bound, which implies lower extreme information loss in the collected data, the 1998 questionnaire was used more than the 1997 questionnaire. We tend to estimate the 1997 responses of individuals to the 1998 questionnaire without modifying the underlying distribution of the answer in mind. The first step is to assume the underlying distribution of cohort at 1997 is $Gamma(\theta _{1997})$ and use MLE to estimate $\hat{\theta }_{1997}=[\alpha =0.801, \beta =2.028]$, then ideal revision matrix

9 $$\begin{aligned} G= \left[ \begin{array}{cccc} 9.44*10^{-1} &{} 1.75*10^{-1} &{} 4.12*10^{-3} &{} 7.88*10^{-7} \\ 2.09*10^{-2} &{} 7.78*10^{-1} &{} 3.25*10^{-1} &{} 8.32*10^{-7} \\ 1.40*10^{-2} &{} 1.25*10^{-2} &{} 5.82*10^{-1} &{} 3.02*10^{-1} \\ 1.19*10^{-2} &{} 1.10*10^{-2} &{} 8.25*10^{-2} &{} 2.89*10^{-1} \\ 8.98*10^{-3} &{} 2.31*10^{-2} &{} 5.31*10^{-3} &{} 4.07*10^{-1} \\ \end{array}\right] \end{aligned}$$

can be computed by $Gamma(\hat{\theta }_{1997})$. The $\alpha$ is the shape parameter and the $\beta$ is the scale parameter of Gamma distribution, when the PDF of Gamma distribution is $f(x)=\frac{1}{\beta ^\alpha \Gamma (\alpha )}x^{\alpha -1}e^{\frac{-x}{\beta }}$.

The next step is to compute the vectors and matrices used in the constraints of VAM, the cohort p.v. of 1997

$$\begin{aligned} u_{1997} = [4.502*10^{-1} \ 2.51*10^{-1} \ 1.17*10^{-1} \ 1.79*10^{-1}], \end{aligned}$$

the cohort p.v. of 1997

$$\begin{aligned} v'_{1997} = [4.307*10^{-1} \ 2.35*10^{-1} \ 1.56*10^{-1} \ 8.57*10^{-2} \ 9.11*10^{-2}], \end{aligned}$$

the transition matrix of 1997

$$\begin{aligned} T_{1997}= \left[ \begin{array}{cccc} 6.86*10^{-1} &{} 3.42*10^{-1} &{} 1.61*10^{-1} &{} 9.15*10^{-2} \\ 1.9002*10^{-1} &{} 3.74*10^{-1} &{} 2.83*10^{-1} &{} 1.24*10^{-1} \\ 7.21*10^{-2} &{} 1.83*10^{-1} &{} 3.13*10^{-1} &{} 2.27*10^{-1} \\ 2.88*10^{-2} &{} 6.13*10^{-2} &{} 1.43*10^{-1} &{} 2.24*10^{-1} \\ 2.23*10^{-2} &{} 3.88*10^{-2} &{} 9.75*10^{-2} &{} 3.32*10^{-1} \\ \end{array}\right] . \end{aligned}$$

Furthermore, the transition matrix of 1998

$$\begin{aligned} B_{1998}= \left[ \begin{array}{ccccc} 6.85*10^{-1} &{} 2.88*10^{-1} &{} 1.42*10^{-1} &{} 9.503*10^{-2} &{} 6.81*10^{-2} \\ 2.001*10^{-1} &{} 4.17*10^{-1} &{} 2.64*10^{-1} &{} 1.26*10^{-1} &{} 7.84*10^{-2} \\ 7.009*10^{-2} &{} 1.94*10^{-1} &{} 3.77*10^{-1} &{} 2.85*10^{-1} &{} 1.12*10^{-1} \\ 2.45*10^{-2} &{} 6.31*10^{-2} &{} 1.42*10^{-1} &{} 2.93*10^{-1} &{} 2.205*10^{-1} \\ 1.93*10^{-2} &{} 3.67*10^{-2} &{} 7.41*10^{-2} &{} 1.99*10^{-1} &{} 5.2004*10^{-1} \\ \end{array}\right] \end{aligned}$$

which does not contain revision effect can also be computed. The third step was to use QP to find a feasible solution of the objective function with constraints (3) to (8) shows, then gave a estimated revision matrix:

$$\begin{aligned} \hat{A}_{1997}= \left[ \begin{array}{cccc} 9.44*10^{-1} &{} 1.75*10^{-1} &{} 4.12*10^{-3} &{} 7.88*10^{-7} \\ 2.09*10^{-2} &{} 7.78*10^{-1} &{} 3.25*10^{-1} &{} 8.32*10^{-7} \\ 1.405*10^{-2} &{} 1.25*10^{-2} &{} 5.82*10^{-1} &{} 3.02*10^{-1} \\ 1.19*10^{-2} &{} 1.101*10^{-2} &{} 8.25*10^{-2} &{} 2.89*10^{-1} \\ 8.98*10^{-3} &{} 2.31*10^{-2} &{} 5.31*10^{-3} &{} 4.07*10^{-1} \\ \end{array}\right] . \end{aligned}$$

Finally, multiplied the p.v. of 1997 $u_{1997}$ with the estimated revision matrix $\hat{A}_{1997}$ gave the revisioned cohort p.v. of 1997

$$\begin{aligned} v_{1997}= [4.69*10^{-1} \ 2.43*10^{-1} \ 1.32*10^{-1} \ 7.0008*10^{-2} \ 8.39*10^{-2}]. \end{aligned}$$

As we assumed the underlying distribution of the cohort at 1997 was Gamma distribution, using MLE to estimate expected value of the cohort by revisioned p.v. of 1997 gave 2.219. Furthermore, the expected value of the cohort in 1997 estimated by the midpoint method was 2.056. In addition, we multiplied the estimated cohort revision matrix $\hat{A}_{1997}$ with population p.v. of 1997

$$\begin{aligned} y_{1997}= [4.91*10^{-1} \ 2.33*10^{-1} \ 1.04*10^{-1} \ 1.701*10^{-1}] \end{aligned}$$

and got revisioned p.v.

$$\begin{aligned} z_{1997}= [5.05*10^{-1} \ 2.26*10^{-1} \ 1.22*10^{-1} \ 6.63*10^{-2} \ 7.97*10^{-2}], \end{aligned}$$

since assuming that the revision effect was the same in population and cohort. By assuming the underlying distribution was Gamma, the estimated annual mean of population was 2.105 by MLE and 1.960 by the midpoint method.

**Fig. 5:**
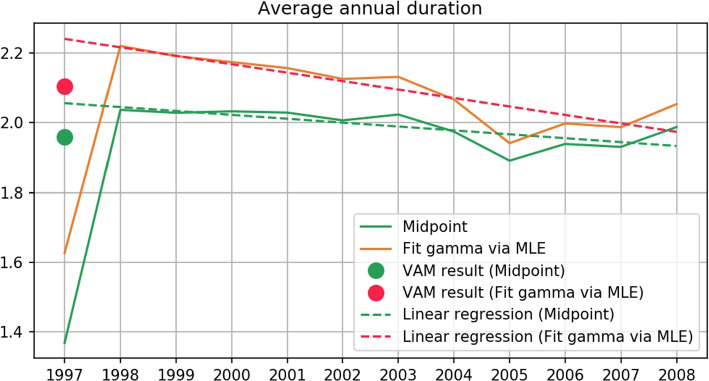
Estimation by VAM and linear regression. This figure depicts the estimation of annual mean in 1997 by VAM with the midpoint method and by VAM with gamma fitting via MLE, which are shown by a green dot and red dot, respectively. Annual means estimated by the midpoint method and MLE from 1998 to 2008 are illustrated by the orange line and green line. Estimated annual means are also fit to a linear regression which is shown by dotted lines, whereas the mean estimation in 1997 is not included

A linear regression (MLE: $R^2=0.766$; Midpoint method: $R^2=0.557$) was fitted to the annual means estimated by MLE and the midpoint method between 1998 and 2008, and it is plotted in Fig. 5. The estimation of annual mean in 1997 by the linear regression is 2.240 via fitting to MLE estimated means and 2.056 via fitting to the midpoint method estimated means. Accordingly, the estimation of mean of 1997 based on VAM revised p.v. (MLE: 2.105; Midpoint method: 1.960) is much closer to linear regression estimation than the estimation based on unrevised p.v. (MLE: 1.626; Midpoint: 1.368). Furthermore, the estimated mean based on VAM revised p.v. lies in the range of estimated means from 1998 to 2008 (MLE: [1.941, 2.220]; Midpoint method: [1.936, 2.057]), which includes no revision effects. In addition to that, the difference between estimated mean of 1997 based on revised p.v. and estimated mean of 1998 (MLE: 0.115; Midpoint method: 0.076) lies in the range of difference of means between consecutive years from 1998 to 2008 (MLE: [0.005, 0.125]; Midpoint: [0.003, 0.083]). On the contrary, the mean estimation based on unrevised p.v. in 1997 induces an irrational annual mean difference between 1997 and 1998 (MLE: 0.593; Midpoint: 0.668). In summary, the estimated mean based on revised p.v. is closer to the estimation by linear regression and lies in the range of means without revision effect in the following years. This implies the estimation is reasonable. The mean estimations by multiple methods mentioned previously are all listed in Table 4.

**Table 4:** Mean estimations based on MJ dataset. The mean estimations of annual LTPA duration in 1997 based on MJ dataset are listed in the column *Estimated 1997 annual mean*. The differences between mean estimation in 1997 and 1998 are listed in the column *Difference of estimated annual means*

	Estimated 1997 annual mean	Difference of estimated annual means
Linear regression (Midpoint method)	2.05624	-0.01118
Linear regression (MLE)	2.24097	-0.02428
Midpoint method	1.36833	0.66870
MLE	1.62673	0.59378
VAM (Midpoint method)	1.96012	0.07691
VAM (MLE)	2.10535	0.11516

The L infinity between revised 1997 p.v. and 1998 p.v. is 2.03%. As it is close to the range of L infinity between consecutive years from 1998 to 2008 ([0.2%, 1.6%]; shown in Table 1 column 5), we concluded that the estimate of response distribution is reasonable. In addition, the unrevised 1997 p.v. have a different sample space of response options, which is that the L infinity between it and 1998 p.v. cannot be computed.

**Fig. 6:**
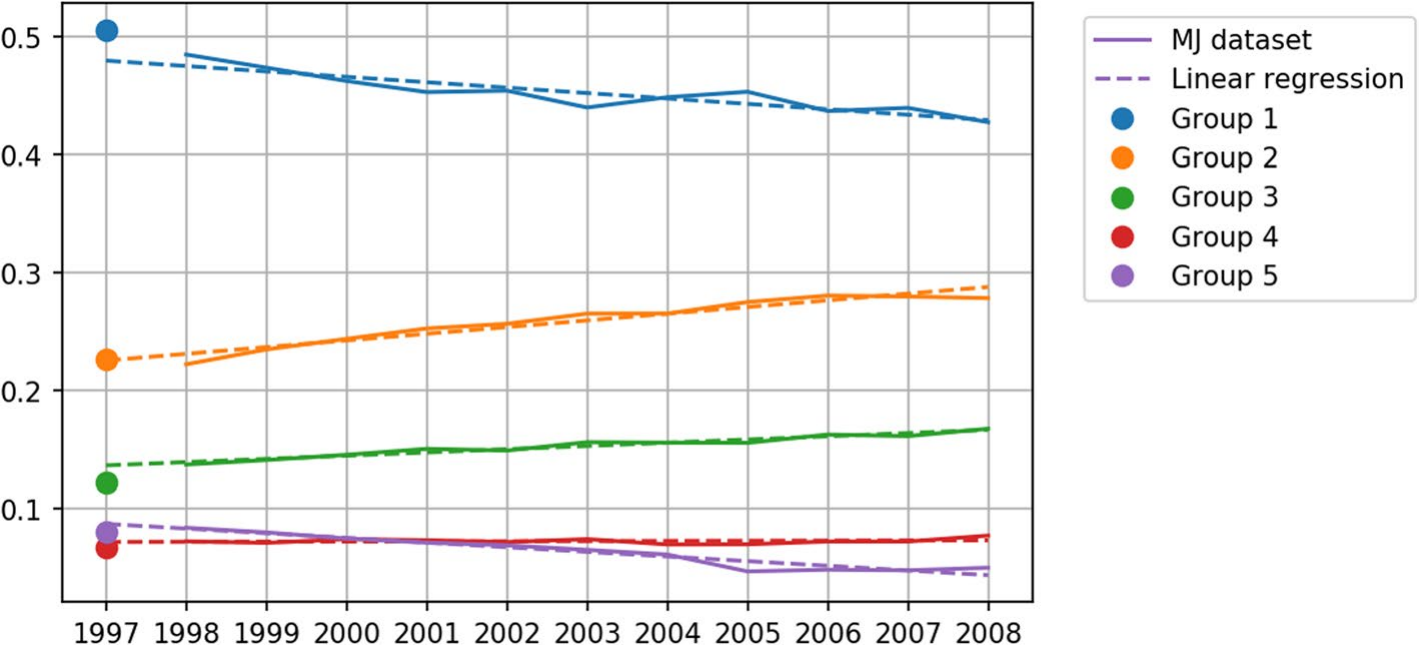
Trend of each group’s relative frequency. The blue, orange, green, red, and purple lines depict the relative frequency of response options 1 to 5, respectively. The dotted line depicts the linear regression fit to the relative frequency of each response option from 1998 to 2008. The dots at 1997 represent the relative frequency estimated by VAM

Assuming that there is a linear relationship between the annual relative frequency of each group and time, multiple linear regression lines ($R^2=[0.825, 0.928, 0.949, 0.037, 0.926]$) fitted to data from 1998 to 2008 can be used to estimate the revisioned 1997 p.v. $z_{1997}=[4.79*10^{-1}, 2.25*10^{-1}, 1.36*10^{-1}, 7.1*10^{-2}, 8.6*10^{-2}]$, which is shown in Fig. 6 by dotted line. The L infinity between revisioned 1997 p.v. estimated by VAM and by linear regression is 1.4%, which is a small value that can infer the feasibility of the estimation by VAM. The $R^2$ of linear regression fitted to each group is close to 1 except group 4, because the relative frequency of group 4 is approximately constant from 1998 to 2008.

### The simulation study

In the simulation study, the expected value of underlying distribution at $t_1$ is 3 and the mean of the 57000 drawn samples is 2.982 (parameters of the simulation are all listed in Table 3). The estimated sample mean is 1.908 and 2.978 by the midpoint method and MLE(Gamma), respectively. And the estimated sample mean of the three simulation datasets by VAM(MLE, Gamma) is 3.017, 3.017 and 3.043. In addition to that, the mean of the cohort at $t_1$ is 2.994, and the estimated cohort mean is 1.901 and 2.981 by the midpoint method and MLE(Gamma), respectively. The estimated mean of the cohort based on the three simulation dataset by VAM(MLE, Gamma) is 3.009, 3.009 and 3.035. All the mean estimations based on simulation dataset are shown in Table 5. All the simulation dataset share the same true values, since the only difference between them is the time-related transition matrix $B_1$. Due to the same reason, the estimation by the midpoint method and MLE(Gamma) based on the three simulation datasets are the same. The estimation result shows that VAM(MLE, Gamma) and MLE introduced a lower estimation error, whereas the estimation error caused by the midpoint method is non-negligible. In addition, the estimation on simulation dataset 1 and 2 are almost identical. Since both of them have similar estimated variable of similarity $\alpha$, which implies that the estimated revision-related matrix $\hat{A_1}$ are similar. Furthermore, due to the scenario of selecting cohort is completely random, revision-related matrix $A_1$ of all the observations is the same as the cohort; hence, the mean of all the observations estimated by VAM is close to the truth. Furthermore, there are two reasons which make the estimation by MLE reasonable. The first one is that the cohort was randomly selected from the samples; thus, the assumption of the underlying distribution on cohort is definitely correct, and the estimation by Gamma fitting via MLE is unbiased. The second reason is that the effect of revision and response bias was not simulated. In addition, the Ideal Survey Response Model does not consider the response bias and the Gamma fitting via MLE does not consider it either. This coincidence makes Gamma fitting via MLE give a reasonable estimation.

**Table 5:** Mean estimations in simulation. The mean estimation of population based on simulation dataset and the true mean values are listed in the column *Sample*. The mean estimation of cohort are listed in the column *Cohort*. The estimation by VAM based on the three different simulation datasets are listed in the last six rows. *Cohort is randomly drawn from samples of population, thus, the underlying distribution of it is identical to population

		Sample	Cohort
True value (Expected value of underlying distribution)		3	3*
True value (Sample mean)		2.98246	2.99449
Midpoint method		1.90892	1.90185
MLE		2.97855	2.98106
Simulation dataset 1	VAM (Midpoint method)	2.71968	2.71013
	VAM (MLE)	3.01784	3.00904
Simulation dataset 2	VAM (Midpoint method)	2.71968	2.71015
	VAM (MLE)	3.01784	3.00904
Simulation dataset 3	VAM (Midpoint method)	2.7375	2.72794
	VAM (MLE)	3.04389	3.03518

Despite the error of estimated mean, we were also interested in the error of revisioned p.v. estimated by VAM. They are all listed in Table 6. L infinity between p.v. of all the observations estimated by VAM and the true p.v. is 0.38%, 0.38% and 0.55% in simulation dataset 1, 2 and 3, respectively. The estimation error of cohort p.v. in L infinity is 0.085%, 0.085% and 0.49% in simulation dataset 1, 2 and 3, respectively. As the L infinity is small, we can infer that the estimation by VAM is reasonable. Moreover, we deduced that even if the probability vectors came from the same underlying distribution, the L infinity between them can still be greater than 0. Thus, we conducted a simple bootstrap analysis to verify our deduction and the feasibility of the estimation by VAM. The underlying distribution was set as Gamma(1,3), the sample size was set to 57000 and 20000, and the number of samples was set to 1000, these parameters were the same as the simulation parameters. By generating two groups of 1000 samples(p.v.), $10^6$ L infinities can be computed between samples in the two groups, then a 95% confidence interval (CI) can be established. The 95% CI of the 57000 sample size is [0.0012, 0.0068], and the estimation error of all the observations by VAM falls in this interval. We can deduce that the estimation error is tolerable. In addition, the 95% CI of the 20000 sample size is [0.00204, 0.0116], and the cohort estimation error of simulation dataset 3 lies in the interval. However, the estimation error of simulation dataset 1 and 2 does not lie in the 95% CI. Since lower estimation error implies smaller distance between estimation and the true value, the estimation of simulation dataset 1 and 2 are reasonable. In summary, the estimation error of VAM can be calculated by L infinity, and it is smaller than 1%, which is negligible. Furthermore, as [15] used KL divergence as the distance measurement method between vectors, we, too, computed the 95% CI in KL divergence. The 20000 sample size 95% CI is [$2.4*10^{-5}$, $5.6*10^{-4}$] and the 57000 sample size is [$8.5*10^{-6}$, $1.9*10^{-4}$]. The estimation error in KL divergence of all the observations is $7*10^{-5}$ and $7*10^{-5}$ based on simulation dataset 1 and 2. They all lie in the 95% CI. However, the estimation error based on simulation dataset 3 do not lie in the 95% CI, thus a drift in the parameter of the underlying distribution in the bootstrap analysis is performed for measuring the amount of the error. When the parameter of one of the underlying distributions is drifted to Gamma(1, 3.02) and the other remains to be Gamma(1, 3), the 95% CI change to [$10^{-5}$, 0.00024]. As the drifted 95% CI includes the estimation error of simulation dataset 3, we deduced that the estimated p.v. is more likely to be drawn from Gamma(1, 3.02). Since the amount of drifting is small, the estimated p.v. is feasible. The estimation error in KL divergence of the cohort is $5*10^{-6}$, $5*10^{-6}$ and 0.00017 based on the simulation datasets. They are reasonable, as all of them lie in the 95% CI or smaller than the lower endpoint of the 95% CI. According to the bootstrap analysis above, distances between probability vectors drawn from identical distribution are not 0. Moreover, the distribution of those distances can form a 95% CI, representing a range in which most of the distances appear. Most of the estimation errors from VAM in L infinity and KL divergence lie in the 95% CI. We can deduce that the estimation error is reasonable, as their underlying distribution can be proved to be the same by bootstrap analysis.

**Table 6:** Estimation error of probability vector by VAM in simulation. The 95% CI is computed by sampling from identical distribution (Gamma(1, 3)) and compute the distances between the samples. The estimation error of population are listed in the column *Sample*. The estimation error of cohort are listed in the column *Cohort*. The estimation error based on the three different simulation dataset are listed in the last six rows

		Sample	Cohort
95% CI in L infinity		[0.0012, 0.0068]	[0.00205, 0.0116]
95% CI in KL divergence		$[8.5*10^{-6}, 0.00019]$	$[2.4*10^{-5}, 0.00056]$
Simulation dataset 1	L infinity	0.00384	0.00085
	KL divergence	0.00007	$5*10^{-6}$
Simulation dataset 2	L infinity	0.00384	0.00085
	KL divergence	0.00007	$5*10^{-6}$
Simulation dataset 3	L infinity	0.00557	0.00490
	KL divergence	0.00024	0.00017

## Discussion

We found a serious issue with the grouped data collected in the MJ dataset, which is that the big gap of estimated annual mean occurs when the revision happens. The method that a previous study [5] suggested also did not consider the revision effect and caused the same issue. We proposed that there are some systematic errors included in the estimation result, which were different when different versions of the questionnaire are used to collect the grouped data. Hence, when conventional methods are used to analyze the data collected by different versions of the questionnaire, the result is unreasonable and includes unexpected errors. According to the result of the MJ dataset, the systematic error corresponding to the loss of information in the underlying distribution and the group with infinite upper bound caused MLE to have a more severe underestimation on the mean of grouped data collected by the 1997 questionnaire than the data collected by the 1998 questionnaire. However, in the simulation study, there is no obvious gap between MLE’s estimation on the grouped data collected by the 1997 questionnaire and by the 1998 questionnaire, which means that there are some differences between the simulation by the proposed Ideal Survey Response Model and the underlying survey response scenario in the MJ dataset. As the simulation study referred to the Survey Response Model defined earlier, which assumed an ideal response process, we inferred that irrational responses or response bias may be one of the reasons that cause the difference; for example, respondents will tend to avoid answering an option with an extreme value [3]. As the response bias is not the same in different versions of a questionnaire, the systematic error induced is different and the estimated mean is inconsistent.

### Future research

VAM has taken all the effects caused by revision into account, which included information loss and response bias, and finally estimated a revision matrix which represents the revision effect. The implicit meaning and structure of the revision matrix is still under study. Further research can focus more on the pattern of response bias in real survey responses, and adjust the Survey Response Model by considering its effects, which can make the simulation closer to real survey response scenarios.

Some conditions need to be satisfied before using VAM. First, a sufficient cohort size is required to eliminate errors that occur when estimating cohort transition matrices. Then, an assumption that the cohort and the population have similar behaviors on revision is required, which implies that the population and cohort revision-related matrices are similar. Future studies can examine whether the revision matrix of different grouped data describing the same behavior shares the same value or not.

The core of VAM is using different transition matrices to denote different transitions related to different factors that affect the distribution of survey responses. The order of the transition matrices is arbitrarily decided in this study: we assumed the revision-related transitions first affect p.v. and then the time-related transitions affect p.v. In the scenario of the MJ dataset, the order of the transitions we used made the decomposed time-related matrix have the same dimension as the transition matrices in the future, which does not include the effect of revision. The revision occurs between 1997 and 1998, so the transition matrices between 1998 to 2008 can be estimated, and it only consists of time-related transitions. Therefore, the transition matrices in the future can be a reference to the current time-related matrix. In the MJ dataset, there were no observations before 1996, and the 1996 questionnaire was not a grouped data type scale; thus, the transition matrix between 1996 and 1997 can not be estimated. Thus, if we use the opposite order of the matrices, the dimension of the decomposed time-related matrix is the same as the transition matrix before 1997. Then, there is no reference to the time-related matrix during the VAM estimation, the estimation error becomes higher and the estimation becomes unreasonable. In the future, when using VAM in other datasets with complete observations where the transition matrices before and after revision can be estimated directly, another order of the transition matrices can be considered, and comparing the results of different orders will be interesting to study.

In this study, the assumption to the underlying distribution is fixed to Gamma distribution, because it has the greatest likelihood computed via MLE. [4] proposed that the underlying distribution of a grouped data can be multimodal. The PDF can be composed of multiple linear density functions of each group, and the response corresponding to the upper limit option can be assumed to follow a Pareto distribution. However, the problem is that it will generate a discontinuous density function; thus, a comparison of estimation obtained by the method we proposed and the method [4] proposed can be an interesting task for the future.

The distance measurement method we used in VAM was L infinity, as we assumed that when the maximum relative frequency difference is small enough, the p.v. and transition matrices were similar. However, L infinity does not take into account the difference of the shape of distribution; hence, only when L infinity is close to 0, the difference of distributions can be considered as negligible errors. In contrast, when L infinity is not close to 0, it may not be a proper measurement method, because greater L infinity does not guarantee a greater dissimilarity. Accordingly, it is reasonable to use KL divergence on p.v.’s distance measurement, as it considers the difference of the entire distribution. However, KL divergence has some disadvantages; for example, it doesn’t satisfy the commutative property and the outcome is an undefined value when some cell in p.v. is 0. It will be interesting to study other measurement methods in the future.

The generalizability of VAM has not been verified because the simulation process and most simulation parameters were referenced from the MJ dataset. As noted earlier, there were some differences between the simulation dataset and the MJ dataset, which can be observed by the estimation of MLE. The reason was that the revision matrix that generates in the simulation is ideal, which means that it did not contain some systematic errors that occurred in the MJ dataset. The method of extracting the transitions that cause these systematic errors from the revision matrix can be studied in the future.

We believe that when designing the grouped data type scale, increasing the number of options and narrowing the interval of each option may be able to reduce the systematic error. More research still needs to be conducted to find the best number of options and the best interval of each option. In addition, the option with an infinite upper bound can add extra space for respondents to answer their actual value, which may reduce the systematic error due to lower information loss.

### VAM with various grouped data as input

Many grouped data do not share the same scenario with the MJ dataset. Some scenarios make estimation by VAM more accurate, but others can make a worse estimation or even make VAM unavailable. Fortunately, by making some additional assumptions, VAM can still estimate the unacceptable data. We list some of the scenarios below, and provide suggestions on estimation for the data that cannot be analyzed by VAM.

There is a type of data where all observations at revision are included in the cohort. Thus the assumption that cohort and all the observations have the same revision behavior can be discarded, which makes the estimation result more accurate. Another type of data, which contains similar cohorts in every year, improves the estimation of VAM. Given that the observations at $t_1$ collected by $Q_1$ need to be revisioned and the observations at $t_2$ are collected by $Q_2$. Assume the demographic of the two cohorts at $t_1$ and $t_2$ are similar, the transition matrix $B_1$ and $B_2$ estimated by them is similar, because they have similar behavior after reading the health screening report, then the L infinity between the two transition matrices is close to 0. Furthermore, assume the demographic between cohorts at $t_2$ and $t_3$ are also similar, which means that $B_2$ and $B_3$ are similar. As $B_1$, $B_2$, and $B_3$ are estimated from the cohorts with similar demographic, the L infinity of $B_1$ and $B_2$ is close to L infinity of $B_2$ and $B_3$, this relationship improves the rationality of referencing L infinity between different time-related transitions matrices.

On the other hand, when the dataset does not include any cohort information, the cohort transition matrix cannot be estimated directly, VAM can not revise the inconsistent observations. Then MLE can be used because it is the most robust method to analyze grouped data, and the estimation error is lower when the number of groups are large and relative frequency of the infinite boundaries are not high. Another situation is where the dataset includes cohort information, but only the cohort information at the target revision time is missing; then, VAM is not applicable to this dataset. A solution is to assume that the time-related transition matrix is similar between the time that revision occurs and the consecutive time without revision. Then, multiply the transition matrix with the p.v. to obtain the estimated revisioned p.v. If the p.v. that we want to reduce the revision effect does not have compatible dimension with the referenced transition matrix in the consecutive time, then the transition matrix needs to be inverted. The inverse matrix may contain negative numbers and may not satisfy the basic property of transition matrix, and the estimated p.v. may not satisfy the basic property of the probability vector either, then MLE is the proper method. Another type of dataset only contains cohort at the time $t_1$ when revision occurs and another time $t_2$ without revision, then assuming $T_1$ and $T_2$ are close is a choice. Accordingly, shrinking the variable $\beta$ to the smallest value possible in the estimation procedure of VAM is required, and the estimation error may be greater as there is no reasonable reference of the distance between $T_1$ and $T_2$. In a more extreme situation where the dataset only contains cohort at the time $t_1$ that revision occurs, then VAM is not available, the MLE is a proper method.

### Limitations

One limitation of this study is that we only use one dataset. The long-term time series data is difficult to obtain; hence, VAM did not apply to various datasets to verify its performance, and the generalizability of VAM was not realized. Another limitation is that the MJ dataset did not have valid data before 1997, which limited the order of matrices after decomposition. Thus, if a complete dataset is made available, then another order of matrices after decomposition can be considered, and the performance of both matrix orders can be compared.

## Conclusion

Using the proposed VAM, we modeled questionnaire revision as transitions between stochastic processes to align the revision-related difference in responses and reduce the inconsistency introduced by it. Our findings are important in the longitudinal study with a long length grouped data type scale, which has high probability to include a revision. Additionally, the inherent information loss in grouped data type scale is not the only factor that causes revision effect. Response bias is another factor that causes the revision inconsistency. Examining the separate effects caused by the two factors is an interesting future research direction.

## Supplementary Information

**Additional file 1.** Data and estimation in the simulation study.

## Data Availability

Some data (simulation dataset) generated during this study are included in this published article and its Supplementary information files. All the codes used in this study are included in this published article and its Supplementary information files. Some data (MJ dataset) that support the findings of this study are available from MJ Health Research Foundation but restrictions apply to the availability of these data, which were used under license for the present study, and hence, are not publicly available. Data are however available from the authors upon reasonable request and with permission of the MJ Health Research Foundation. Researchers interested in access to the data and code may contact Chung-Han Liang at r09922a02@ntu.edu.tw.

## Abbreviations

MLE: Maximum likelihood estimator
p.v.: Probability vector
VAM: Version alignment method
QP: Quadratic programming
PDF: Probability density function
CDF: Cumulative distribution function
CI: Confidence interval
